# Monkeypox Virus: Epidemiology, Virology, Diagnosis, Vaccine, and Therapeutics

**DOI:** 10.1002/mco2.70525

**Published:** 2026-01-02

**Authors:** Yunzheng Yan, Yaqin Sun, Guangyan Sun, Cheng Niu, Xinyuan Zhao, Ming Zhao, Tongyao Liu, Suyue Zhang, Hui Zhai, Ankang Liu, Shouzhi Yu, Shuyuan Pan, Wu Zhong, Yuntao Zhang, Song Li

**Affiliations:** ^1^ State Key Laboratory of Novel Vaccines for Emerging Infectious Diseases Beijing Institute of Biological Products Company Limited Beijing China; ^2^ National Engineering Research Center for the Emergency Drug Beijing China; ^3^ State Key Laboratory of Novel Vaccines for Emerging Infectious Diseases China National Biotec Group Company Limited Beijing China

**Keywords:** diagnosis, epidemiology, monkeypox virus, therapeutics, vaccine, virology

## Abstract

Since 2022, mpox epidemics have been sustaining and escalating over the world, posing a significant public health challenge. While significant progress has been made in diagnostic methodologies, prophylactic vaccines, and therapeutic interventions to mitigate monkeypox virus (MPXV) infection, scientific understanding of MPXV and related orthopoxviruses continues to evolve progressively. In order to keep pace with recent advancements, herein we review progress in mpox research from five key perspectives. This article first summarizes the latest epidemiological profiles, incorporating different viral lineages globally and in China, while highlighting their evolutionary history and distinct clinical characteristics. The virological profiles of MPXV shed light on its complete infectious lifecycle and the formation of distinct virus particle types. Clinically approved classical detection methods and emerging novel testing techniques are provided, establishing a framework for early diagnosis of mpox patients. The efficacy and safety of both licensed vaccines and those under development are analyzed to underscore their value in preventing mpox infection. Additionally, progress in approved and newly identified potential therapeutic agents is summarized and discussed, aiming to provide insights for further drug development and clinical treatment strategies.

## Graphical abstract

This review provides a comprehensive overview of the latest research advances in MPXV, with a focused analysis across five core dimensions: epidemiology, virology, diagnosis, vaccine, and therapeutics. The primary objective of this review is to advance the understanding of MPXV and facilitate the development of more effective diagnostic methods, vaccines, and treatments for the control and mitigation of mpox.

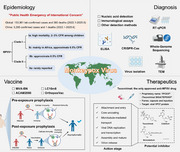

## 1 Introduction

Monkeypox virus (MPXV) is a double‐stranded DNA virus belonging to the genus *Orthopoxvirus*, the family Poxviridae [1]. The disease caused by MPXV, namely mpox, is a zoonotic viral disease with smallpox‐like clinical manifestations [2]. MPXV can infect humans via various routes, such as the skin, respiratory tract, and mucous membranes under conditions of close human‐to‐human contact or environmental exposure; the specific transmission route significantly affects the duration of the incubation period [2]. In addition, virus strain, exposure route, infection dose, and the host's immune status all have significant impacts on the clinical presentations. Before skin lesions appear, the majority of patients exhibit prodromal symptoms such as fever, lymphadenopathy, myalgias, malaise, and headache; subsequently, the classic development process of the skin lesions, from the stages of macules, papules, vesicles, and pustules to scabs, could resolve within 2–4 weeks [3, 4, 5, 6]. It is noteworthy that immunocompromised populations (HIV‐infected individuals, children, etc.) account for a high proportion among mpox patients, increasing susceptibility to severe disease and the risk of death [7, 8, 9, 10].

Since the 1970s, mpox has emerged or reemerged in Central and West Africa, posing a significant threat to public health and safety [11]. Unprecedentedly, a global mpox outbreak began in May 2022, and the World Health Organization (WHO) declared it a “Public Health Emergency of International Concern (PHEIC)” in July 2022 and August 2024, respectively [12, 13]. On August 12, 2022, the WHO classified MPXV into two major clades: Clade I (formerly Central African clade) and Clade II (formerly West African clade) [14]. Clade II was further subdivided into subclades IIa and IIb [15]. In April 2024, Clade I was further subdivided into Clade Ia and Clade Ib following the detection of genetically distinct variants [16]. Notably, these two global outbreaks were driven by distinct MPXV strains, with significant differences in demographic distribution, disease severity, and clinical course [17]. For instance, the Clade IIb‐driven epidemic predominantly affected male populations, particularly men who have sex with men (MSM). Clinical manifestations included genital/perianal lesions, fever, and lymphadenopathy, with the most frequent number of skin lesions ranging from 2 to 10 [18, 19]. In contrast, Clade Ib infections are associated with more severe phenotypes: the median maximum number of skin lesions in Clade Ib patients reaches 60 [20], reflecting a more acute and aggressive clinical course.

To provide a comprehensive understanding of MPXV and mpox, this review first updates on recent advancements in epidemiological profiles from the perspective of different strains, both globally and in China. We also outline the virological characteristics of MPXV, including its morphology, genome, and life cycle. Additionally, we systematically summarize and analyze progress in mpox diagnosis, vaccination, and therapeutic strategies, along with perspectives on future research directions. Together, these efforts aim to inform the prevention and control of mpox by offering valuable insights.

## 2 Epidemiology

MPXV was first identified in cynomolgus monkeys in 1958 [21]. In 1970, scientists isolated MPXV from a smallpox‐like patient in the Democratic Republic of the Congo (DRC; formerly Zaire, 1971–1997), marking the first recognized human mpox case in medical history [22]. Since then, mpox has remained endemic in Africa, and after 2003, it has gradually spread globally [21], including to China. Between January 1, 2022 and September 30, 2025, 140 countries reported 168,974 laboratory‐confirmed mpox cases and 441 deaths to the WHO, concurrently with the evolution of distinct viral lineages [23]. Notably, dominant circulating strains have varied temporally and geographically, differing in susceptible populations and mortality rates. This section details the global and Chinese epidemiological profiles of these MPXV clades.

### 2.1 Global Epidemiology of Mpox

Following its initial detection in Zaire, mpox remained endemic in Central and West Africa for decades. Between 1970 and 1997, over 700 confirmed mpox cases were reported in Africa, primarily in Zaire, with case fatality rates (CFRs) as high as 9.8%–17% [24, 25, 26]. Phylogenetic analyses later attributed these outbreaks with high mortality to MPXV Clade I; prior to 2022, the overall CFR of Clade I was approximately 10.6% [27]. Historically, Clade I infection presented the following clinical features: prodromal fever followed by a characteristic monomorphic, centrifugal rash often initiating on the face [28, 29].

Notably, in 2003, the first mpox case outside Africa was reported in the United States, which was linked to imported animals from Ghana, marking the onset of global spread of mpox [30, 31]. An analysis of 35 cases from this outbreak found that 85% of them presented with prodromal fever or fever with lesions; more than 77% had respiratory symptoms; 69% exhibited lymphadenopathy; and lesions were distributed on the head, trunk, arms, and legs [17]. This outbreak was confirmed to be caused by MPXV Clade IIa [17, 32]. To date, Clade IIa remains primarily associated with sporadic zoonotic transmission via human‐animal contact, with an overall CFR of approximately 1% as of 2023 [33].

Clade IIb first emerged in 2017–2018 in Nigeria, causing an outbreak with 122 confirmed and suspected cases [34]. Subsequent phylogenetic analyses further defined the clade associated with this outbreak as Lineage A [35, 36]. Patients presented with cutaneous rashes on the face, legs, trunk, and genitals, accompanied by fever and lymphadenopathy [34]. Subsequently, Lineage B.1 of Clade IIb triggered a global outbreak in 2022 [36]. By July 2022, over 16,000 cases had been reported to the WHO from 75 countries/regions, prompting the first declaration of mpox as a PHEIC [37]. Characterized by exclusive human‐to‐human transmission, this outbreak predominantly affected MSM, including gay and bisexual populations [38]. The mean incubation period ranged from 2 to 21 days [38], with primary symptoms such as cutaneous rashes, fever, headache, and lymphadenopathy. Symptoms typically resolved within 2–4 weeks, but persisted longer in immunocompromised individuals [16]. Unlike prior outbreaks, this Clade IIb manifestation was relatively mild, with rashes mainly on the genital region [39, 40]. Although reported cases have since plateaued, Clade IIb transmission persists globally, with an overall CFR of approximately 0.1% as of 2023 [40]. A recent study indicated that countries with robust public health systems reported fewer mpox cases and deaths [41].

In 2024, a pandemic driven by MPXV Clade I broke out in the DRC and subsequently spread to neighboring countries as well as non‐African regions (e.g., Sweden, the United Kingdom, Thailand) [42]. This resurgence prompted the WHO to redeclare mpox a PHEIC in August 2024 [13]. Distinct from Clade IIb, Clade Ib outbreaks involve a significant proportion of women and children [13]. Clinically, a study of 226 suspected Clade Ib cases in South Kivu Province, DRC, revealed universal cutaneous rashes, with 59% developing fever, 40% exhibiting lymphadenopathy, and nonspecific symptoms (e.g., headache, myalgia) in patients [43]. Prospective studies also confirm that Clade Ib infection is associated with generalized rashes, fever, lymphadenopathy, and a notable risk of fetal loss during pregnancy [17, 43, 44, 45]. From January 2024 to October 24, 2025, 41 countries reported Clade Ib cases to the WHO, mostly concentrated in Africa [23].

**FIGURE 1:**
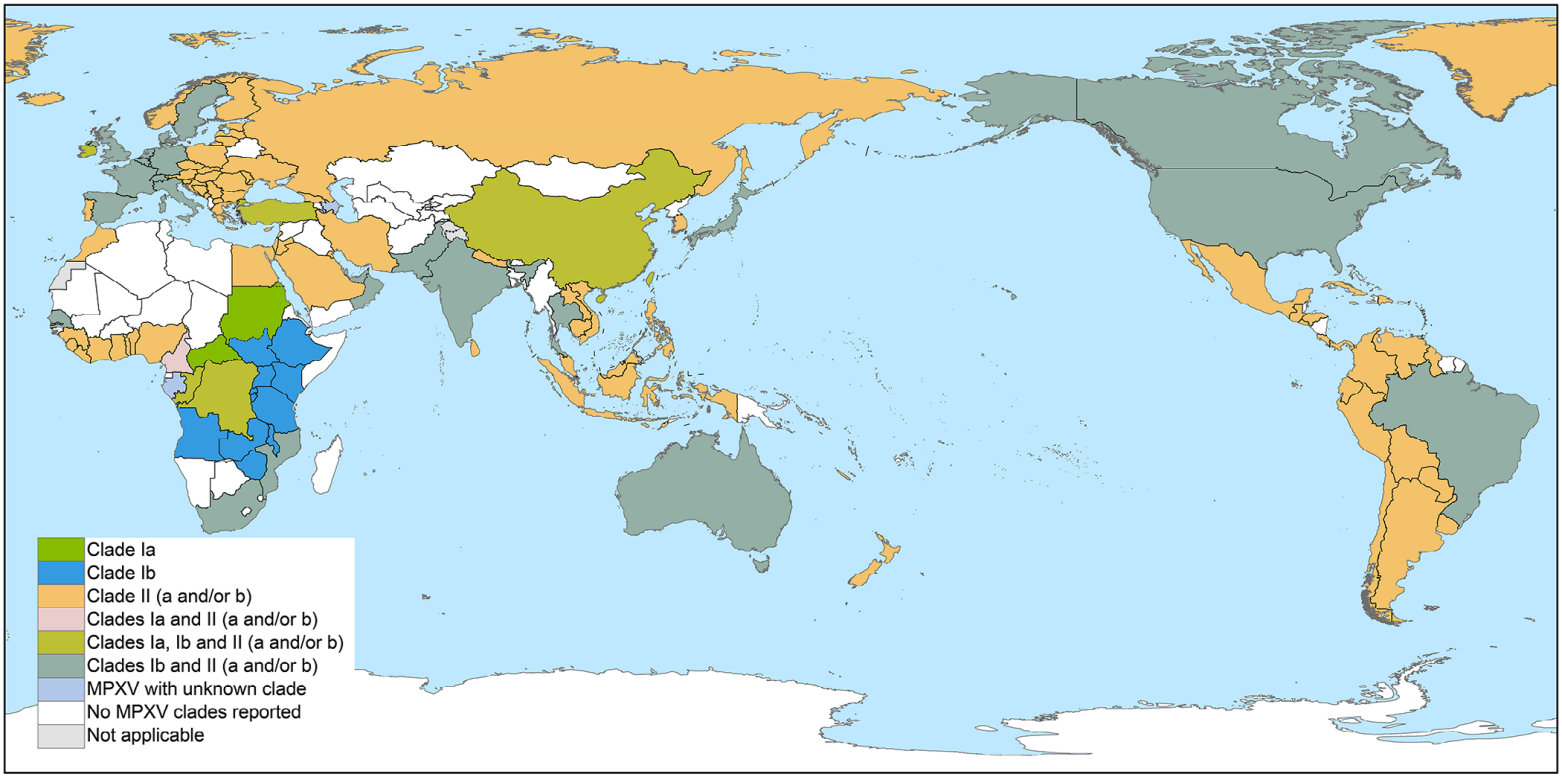
Geographic distribution of MPXV clades reported to WHO, from January 1, 2022 to October 5, 2025. Clade II (encompassing sublineages a and/or b), represented by the orange color, shows extensive spread across multiple continents‐including large portions of North America, South America, Europe, parts of Asia, and Oceania. Clade Ib has also spread widely, encompassing Africa, most of North America, parts of South America, parts of Europe, parts of Asia, and Oceania. In contrast, Clade Ia (green) is largely confined to central Africa. Additionally, regions in light pink, light orange, light green (e.g., combinations of Clades Ia, Ib, and II) reflect areas where multiple MPXV clades co‐circulate. Areas in white indicate no reported MPXV clades, and light blue denotes cases with unknown clade classification. (Data *Source*: WHO website, https://worldhealthorg.shinyapps.io/mpx_global/).

In July 2025, the WHO released its latest epidemiological update clarifying the global clade landscape: Clade Ia remains endemic in the DRC with persistently high mortality, particularly among children (2%–3% CFR); Clade Ib circulates predominantly in Africa, with travel‐associated cases in other regions and an approximate 0.5% CFR; Clade IIa is rarely reported; and Clade IIb maintains stable transmission in non‐African regions (500–1,000 monthly cases; ∼0.5% CFR), but recently triggered an outbreak in Sierra Leone (African region) [46]. Notably, actual cases may be underestimated due to potential underreporting caused by diagnostic and surveillance incapacities. The global distribution of mpox clades is illustrated in Figure 1.

### 2.2 Epidemiology of Mpox in China

Mainland China reported its first imported mpox case in September 2022 [47]. The first indigenous case emerged in June 2023, followed by a surge peaking in August 2023; all cases during this surge were attributed to Clade IIb [48]. An analysis of epidemiological characteristics (June–December 2023) identified 1,702 male cases (99.42%) and 10 female cases (0.58%), with a median age of 31 years (range: 15–71 years). Approximately 90% of cases reported same‐sex male sexual contact within 21 days prior to symptom onset, consistent with global transmission patterns. Among 1,666 tested individuals, 42.56% (*n* = 709) were HIV‐positive [48]. A multicenter observational study (*n* = 286, September 2022–October 2024) further characterized the affected population, identifying MSM aged 27–37 years in coastal and economically developed regions as the primary group. This study also demonstrated that HIV coinfection was associated with severe disease, including elevated AST/CRP levels and reduced CD4^+^ T cell and NK cell counts [49].

In January 2025, the China Center for Disease Control and Prevention (CDC) reported a Clade Ib outbreak traced to a returnee from the DRC, resulting in four secondary cases through close contact [50]. One secondary case was a female presenting with limb pustules and trunk/buttock lesions, without fever or genital rash [51]. This clinical presentation differed from typical Clade IIb cases, which commonly feature fever, lymphadenopathy, and genital involvement [52, 53]. As of October 24, 2025, China had reported 29 Clade Ib cases to the WHO [23]. Additionally, China reported travel‐associated Clade Ia cases to the WHO in 2025 [23].

To date, mpox cases infected with Clade I and Clade II have been documented in China. The WHO statistics indicate 3618 confirmed mpox cases and one death (July 2024) in China between January 2022 and September 2025 [23]. It is notable that effective vaccines and treatments are urgently needed to combat potential outbreaks of mpox, while dozens of mpox cases are reported per month in China currently.

## 3 Virology

**FIGURE 2:**
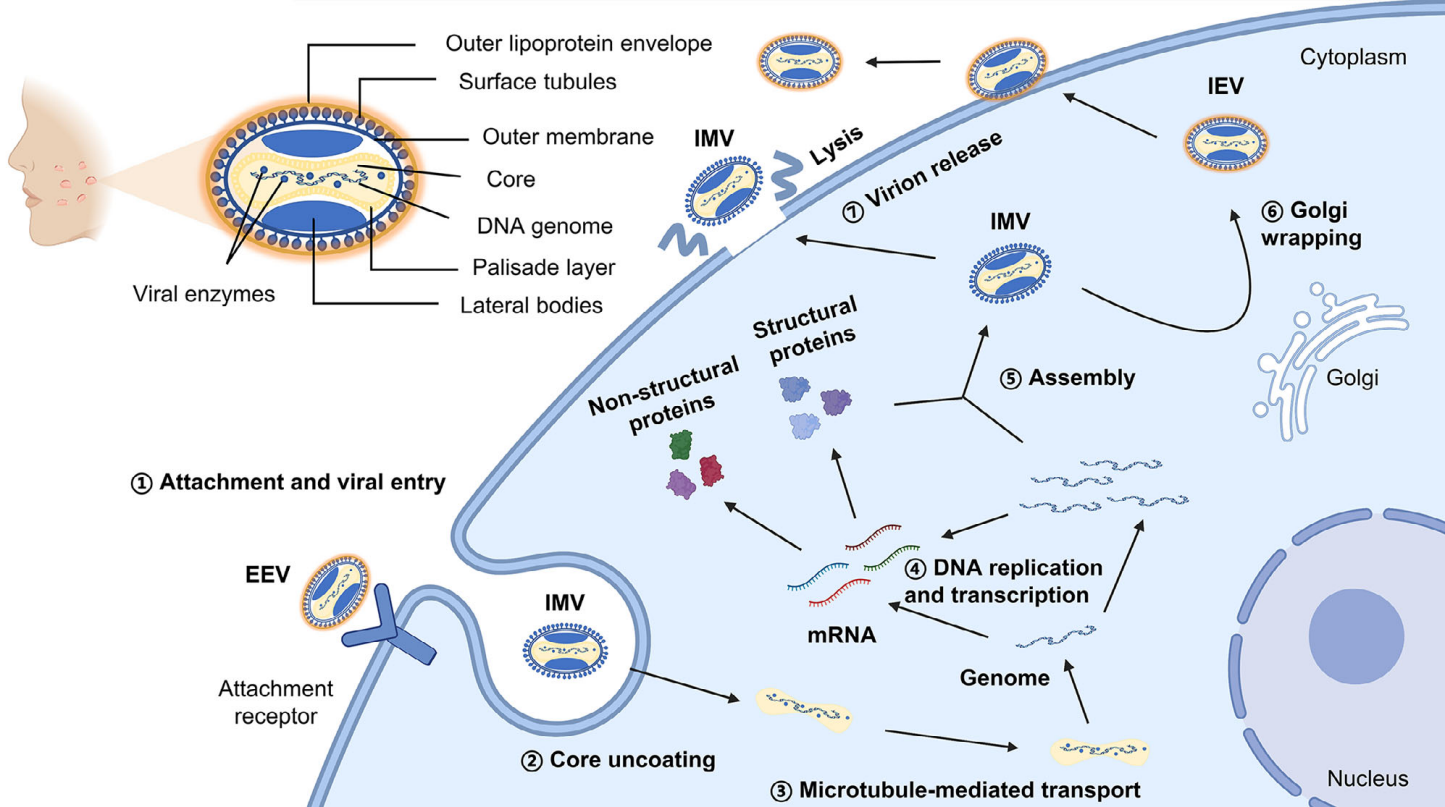
The viral structure and life cycle of MPXV replication in host cells. The mpox virion consists of four major components: the core, lateral bodies, outer membrane, and outer lipoprotein envelope, with the core surrounded by a palisade layer and the outer membrane decorated with surface tubules. Its replication cycle proceeds through sequential stages: attachment and entry, uncoating, DNA transcription and replication, viral protein synthesis, virion assembly, and release. Intracellular mature virions (IMV) are either released directly via cell lysis or acquire additional envelopes from the Golgi apparatus to form intracellular enveloped virions (IEV). IEV are secreted either as cell‐associated enveloped virions (CEV), which remain attached to the cell surface, or as extracellular enveloped virions (EEV).

MPXV exhibits close relationships with other members of the *Orthopoxvirus* genus, such as vaccinia virus (VACV) and variola virus (VARV), sharing conserved lifecycle features and similar morphological characteristics. An in‐depth understanding of MPXV virology is crucial for elucidating its replication strategies, immune evasion mechanisms, as well as informing the development of effective vaccines and antiviral agents. This section systematically reviews the fundamental virological characteristics of MPXV, including virion morphology, genome organization, and lifecycle (Figure 2).

### 3.1 Morphology

VARV shows a brick‐shaped architecture of roughly 250–350 × 200 nm as determined by diagnostic electron microscopy [54]. Furthermore, recent advances using cryo‐electron tomography have demonstrated that the majority of mature MPXV particles exhibit an oblate ellipsoidal shape with dimensions of approximately 313 × 267 × 236 nm, in contrast to the more rectangular morphology of VACV particles (around 347 × 260 × 240 nm), with statistically significant differences in both the long and intermediate axes [55]. Structurally, these virions consist of four major components: core, lateral bodies, outer membrane, and outer lipoprotein envelope [56, 57].

The core, which appears as a biconcave structure under electron microscopy, contains the linear DNA genome, core fibrils, essential viral enzymes, and associated transcription factors [58, 59, 60]. Surrounding the core, there is a dense, rod‐like structural layer known as the palisade layer [61], composed of A10 trimers with dimensions of ∼8 × 13 nm (width × height) [55]. MPXV possesses two lateral bodies, located symmetrically on either side of the core [60]. Following fusion between the viral envelope and host cell membrane, lateral bodies detach from the viral core to deliver effector proteins into the host cytosol, while the released core functions as an early transcription factory [55].

The outer membrane comprises a 50–55 nm lipoprotein bilayer (cholesterol and phospholipid) and its surface displays randomly distributed tubules (average 7 nm in width and 100 nm in length), forming a characteristic textured morphology [57, 62]. The cholesterol and phospholipid content of the bilayer, as well as the existence of surface tubules, are important to maintain the integrity of the outer membrane structure [63]. The virion, composed of a core, lateral bodies, and an outer membrane, is infectious; however, under specific infection conditions such as those involving extracellular enveloped virions (EEV), the particle acquires an additional, structurally distinct lipoprotein envelope [57]. In 1969, Boulter first elucidated the significance of the poxvirus envelope, demonstrating that its absence abrogates the protective efficacy of inactivated poxvirus vaccines upon challenge [64]. Subsequent studies confirmed that envelope‐associated viral antigens would elicit immune responses across systems, conferring effective protection against poxvirus infection [65, 66, 67].

### 3.2 Genome

MPXV has a linear genome with a length of ∼197 kbp [68, 69]. In comparison, VACV and VARV possess genomes of 165–199 kbp [70] and ∼186 kbp [71, 72], respectively. Despite these differences in size, all three genomes contain two functional regions, specifically including a highly conserved central coding region and variable regions on both sides [69, 73]. In addition, both termini of the genome are characterized by inverted terminal repeats, which include the hairpin loop, NR1 and NR2, short tandem repeats, as well as several open reading frames (ORFs) [69, 74]. The above terminal genes play a vital role in regulating host immune responses to achieve immune evasion [75]. The ORFs in the central conserved regions share at least 90% sequence identity with those of other orthopoxviruses, which primarily encode viral proteins involved in the process from viral replication to particle release [69].

### 3.3 Life Cycle

Among orthopoxviruses, VARV's sole natural reservoir is humans; however, MPXV and VACV possess broad natural host ranges, with MPXV mainly infecting primates and rodents [76] and VACV lacking a clearly defined reservoir but possibly involving rodents and marsupials [77, 78]. The MPXV genome encodes approximately 190–200 viral proteins, including structural and nonstructural proteins, which aid in viral replication and the entire lifecycle [69, 79, 80]. There are two kinds of infectious viral particles of poxviruses: EEV and intracellular mature virions (IMV) [81]. Distinct from the IMV's single‐membrane structure, EEV has a double‐membrane structure, and its surface possesses different proteins and membrane components. As a result, these two types of viral particles rely on different modes to achieve viral attachment, hemifusion, and core entry [82]. Additionally, due to the absence of the lipid membrane layer, IMV exhibits more stable properties, which facilitates transmission between different individuals, whereas EEV is considered to mainly play a role in intercellular transmission [83]. Although MPXV is a DNA virus, its replication process is completed in host cells’ cytoplasm rather than in the nucleus.

The initial step of MPXV infection is attachment, primarily mediated by interactions between viral surface proteins and host cell glycosaminoglycans (GAGs) [76, 84]. Although specific cellular receptors for poxviruses remain incompletely characterized, studies on the closely related VACV have revealed multiple viral proteins involved in viral attachment [85]. In MPXV, proteins E8, A29, A28, and H3—homologous to VACV D8, A27, A26, and H3, respectively—play essential roles in mediating attachment [86, 87, 88, 89, 90]. Following attachment, the virus proceeds to the membrane fusion stage, a process mediated by the highly conserved Entry Fusion Complex (EFC) in poxviruses, which comprises proteins ranging from 4 to 43 kDa [82, 91, 92]. The EFC contains at least 11 components, including A16, A21, A28, F9, G3, G9, H2, J5, L1, L5, and O3 of VACV [85, 93]. These proteins do not function independently, and currently three bimolecular interactions have been identified: A28‐H2, A16‐G9, and G3‐L5 [85, 94, 95]. For example, the A16‐G9 subcomplex can interact with either the viral A56‐K2 complex or the A26 protein on the surface of mature virions to inhibit fusion [96]. Upon membrane fusion, the viral core is released into the cytoplasm, where EFC‐associated proteins mediate its uncoating to initiate viral biosynthesis [85, 97]. The exposed viral core is transported to the periphery of the cell nucleus through microtubule structures [76, 98].

Subsequently, the viral genome, together with enzymes as well as regulatory factors, is released from the core of virions, which enables the synthesis of early mRNAs within minutes postinfection [99]. The replication region called the factory can be detected within 2 h postinfection. Multiple viral proteins are involved in poxvirus DNA replication, such as polymerase, helicase – primase, uracil DNA glycosylase, protein kinase, and so on [100]. Furthermore, the transcription system of poxviruses is highly complicated, and this process is regulated in a cascade mechanism involving transcription initiation at early, intermediate, and late stages of infection [101]. In the stages of virus assembly and release, newly synthesized viral components assemble into mature virus particles called IMV [61]. While some IMVs accumulate intracellularly, leading to cell lysis, others traverse the trans‐Golgi network (TGN) or nuclear membranes to form intracellular enveloped viruses (IEV) [102, 103, 104]. IEVs transported to the cell periphery fuse with the plasma membrane via exocytosis, generating cell‐associated enveloped viruses (CEV) [105]. CEV particles either remain surface‐bound or are released as EEV through actin‐ and microtubule‐mediated transport [76, 106, 107].

## 4 Diagnosis of Mpox

MPXV infection is clinically indistinguishable from other eruptive diseases like varicella [108, 109]. Additionally, coinfection with other pathogens, for example, syphilis or herpes simplex virus (HSV), has been documented in 12.1% of hospitalized cases [110]. Consequently, early diagnosis of mpox is both necessary and challenging. Current diagnostic methods include nucleic acid detection, immunological assays, and other approaches. Herein, we summarize the characteristics of MPXV diagnostic methods from three aspects (sensitivity/specificity, cost/requirement, and application scenarios) (Table 1).

**TABLE 1:** The characteristics of detection techniques for MPXV.

No.	Diagnostic methods		Sensitivity and specificity	Cost/requirement	Applicable scenarios	References
1	Nucleic acid detection	Real‐time quantitative Polymerase Chain Reaction (RT‐qPCR)	High sensitivity and specificity	Requiring thermal cycling equipment and well‐trained personnel	Current gold standard method for MPXV diagnosis	[111, 112]
2	Nucleic acid detection	Digital PCR	Exhibiting potential superior to RT‐qPCR	High cost and long processing time	Viral load evaluation	[113, 114]
3	Nucleic acid detection	Loop‐Mediated Isothermal Amplification (LAMP)	Exhibiting potential superior to RT‐qPCR	Complex design process and low equipment requirements (no thermal cycling equipment)	With potential for application in point‐of‐care (POC)	[108, 111, 112, 115, 116, 117]
4	Nucleic acid detection	Recombinase Polymerase Amplification (RPA)	High sensitivity in partial gene targeting (e.g., *G2R*), but frequent nonspecific amplification occurred	Complex design process and low equipment requirements (no thermal cycling equipment)	With potential for application in POC	[111, 112, 118]
5	Nucleic acid detection	Whole‐Genome Sequencing (WGS)	With the ability to identify specific strains and genetic variations	High reagent cost, high equipment requirements, and high operational requirements	Evolutionary tracking; MPXV mutation analysis	[111, 119]
6	Nucleic acid detection	CRISPR‐Cas	Relatively low sensitivity without DNA amplification technology	Complicated design procedure	With potential for field detection	[111, 112, 120, 121]
7	Nucleic acid detection	Combinatorial approaches	Exhibiting potential superior to RT‐qPCR	With potential for easy operation and without specialized equipment	With potential for POC	[111, 112, 122]
8	Immunological assays	Enzyme‐Linked Immunosorbent Assay (ELISA)	With cross‐reactivity to other orthopoxviruses and limited sensitivity	Requiring Biosafety Level 3 (BSL‐3) facilities and large quantities of samples	With the ability to judge MPXV infection time	[111, 112, 123]
9	Immunological assays	Antigen Rapid Diagnostic Tests (Ag‐RDT)	With usually low sensitivity and specificity	Without specialized equipment	With potential for application in POC	[112, 123]
10	Other detection methods	Virus isolation	Able to distinguish virus strains with different mutations when combined with other technologies	Complex operation, long time‐consuming process, and requirement for BSL‐3 facilities	Virological research	[111, 124]
11	Other detection methods	Transmission Electron Microscopy (TEM)	Able to distinguish orthopoxviruses from HSV, but cannot determine the precise species	Complex equipment and operation	Virological research	[111, 124]
12	Other detection methods	Wastewater‐based Surveillance (WBS)	Detection method not established, needing additional assessment	Complex operation and long time‐consuming process	Assessment of MPXV's spatial and temporal distribution	[125]

### 4.1 Nucleic Acid Detection

RT‐qPCR stands as the gold standard for MPXV nucleic acid detection [126]. To date, multiple RT‐qPCR‐based MPXV detection kits have been approved for clinical use globally. Among these kits, the *F3L* gene is the most commonly targeted region [127]. Beyond target selection, commercial kits share consistent characteristics, as exemplified by four kits approved by China's National Medical Products Administration (NMPA) as of May 2025: a sensitivity of 200 genome copies/mL, an amplification time of 30–40 min, and integration of a dUTP‐UDG enzyme system to prevent carryover contamination from PCR amplicons [128, 129, 130, 131]. These kits prioritize skin lesion specimens (rash swabs or exudates), aligning with the WHO guidelines [126, 132]. The WHO additionally recommends oropharyngeal or anal swabs in the absence of skin lesions [126, 132]. Furthermore, due to the relatively short duration of viremia compared to symptom persistence, the WHO advises against prioritizing blood samples for PCR [132]. Despite its high sensitivity, RT‐qPCR's technical complexity confines it primarily to centralized laboratories [112].

In addition to RT‐qPCR, other nucleic acid amplification‐based technologies are increasingly being explored for MPXV detection. Among these, the most extensively studied include digital PCR and isothermal amplification technologies—such as LAMP and RPA. Digital PCR assays exhibit higher sensitivity than RT‐qPCR, with a limit of detection (LOD) of six copies per reaction and a limit of quantitation (LOQ) of 38 copies/reaction [113]; however, this technology limits clinical translation due to its lengthy protocol [108]. Both LAMP and RPA enable nucleic acid amplification under isothermal conditions, eliminating the need for thermal cycling equipment. LAMP, in particular, holds significant promise for POC applications due to its high sensitivity and operability under isothermal conditions [108, 112]. For instance, an *A27L*/*F3L*‐targeted LAMP assay demonstrates an LOQ of 20 copies/reaction with no cross‐reactivity with 21 other pathogens, including related orthopoxviruses [117]. RPA offers shorter turnaround times (results within 10 min) than LAMP, but exhibits nonspecific amplification, particularly in complex clinical matrices [111].

Furthermore, several emerging technologies that do not rely entirely on nucleic acid amplification principles are being developed for the diagnosis of MPXV, including WGS and CRISPR‐Cas systems [108]. WGS enables definitive MPXV identification, distinguishing it from other orthopoxviruses [119], and facilitates evolutionary tracking; however, its high computational cost restricts use to research settings [133]. CRISPR‐Cas technologies identify MPXV DNA and leverage the endonuclease activity of Cas enzymes for viral DNA elimination [120]. Two notable applications, SHERLOCK (Cas13a‐based) and DETECTR (Cas12a‐based), are recommended for diagnostic use [134, 135]. Despite their rapid detection capability, CRISPR‐Cas technologies exhibit relatively lower sensitivity compared to PCR‐based methods due to their non‐amplification‐based principle.

Combinatorial diagnostic approaches, which integrate multiple technologies, have been developed to enhance diagnostic performance. A recent study combined Multiple Enzyme Isothermal Rapid Amplification (MIRA) with CRISPR‐Cas12b, yielding a single‐tube assay with an LOD of four copies/reaction, 98.5% sensitivity, and 97.0% specificity, with no cross‐reactivity to enterovirus A71 or HSV [136]. Other similar strategies merging MIRA‐Cas13a [137] or LAMP‐Cas12b [138] highlight the potential of next‐generation diagnostic platforms for POC applications. Additionally, workstation‐integrated diagnostic platforms have been deployed for MPXV diagnosis, exemplified by GeneXpert and Dragonfly systems. GeneXpert, a portable rapid diagnostic device, integrates orthopoxvirus‐ and MPXV‐specific PCR assays with a 90‐min turnaround time, demonstrating 98.8% sensitivity and 100% specificity [139]. The Dragonfly system combines power‐free nucleic acid extraction with LAMP, achieving an LOD of 100 genome copies/mL, with sensitivities of 96.1% for orthopoxviruses and 94.1% for MPXV, alongside 100% specificity [140].

### 4.2 Immunological Assay

In addition to nucleic acid testing, immunological assays play a critical role in mpox diagnostics. Early studies utilized ELISA, immunohistochemistry, and immunofluorescence assays for MPXV confirmation [120]. However, ELISA‐based detection of postinfection IgG/IgM is hampered by cross‐reactivity with antibodies induced by prior smallpox/vaccinia vaccination [124], limiting its POC utility [108, 123, 141].

Inspired by COVID‐19 antigen rapid diagnostic tests (Ag‐RDTs), researchers have attempted to develop mpox‐specific antigen or antibody assays. However, cross‐reactivity with other orthopoxviruses poses a significant challenge [112]. Consequently, the WHO currently does not endorse antigen or antibody detection for primary diagnosis. A recent evaluation of three Clade IIb Ag‐RDTs showed an LOD of 1.0 × 10⁴ PFU/mL and 100% specificity but critically low sensitivity (0.00%–15.79% for skin samples, 0.00% for respiratory samples), thereby strongly discouraging their use for diagnosis or screening [142].

Despite these limitations, technologies related to Ag‐RDTs have been advancing continuously. Specifically, assays targeting the A29L antigen have demonstrated promising sensitivity and specificity [143]. For instance, a lateral flow immunoassay (LFIA) based on two hybridoma‐derived anti‐A29L monoclonal antibodies (mAbs) differentiated MPXV from VACV, with an LOD of 7.5–15 ng/mL [143]. Another LFIA achieved an LOD of 50 pg/mL with no cross‐reactivity to orthopoxviruses or respiratory pathogens like SARS‐CoV‐2 or influenza A/B [144]. Additionally, a recent study reported that the pixel‐diverse interferometric reflectance imaging sensor enabled quantification of MPXV infection via targeting the A29 locus, with an LOD of 200 PFU/mL. This platform discriminated between HSV and CPXV, offering POC potential [116]. Antigen detection kits targeting other loci, including the A5L locus, are also under development [145].

### 4.3 Other Detection Methods

Other approaches for MPXV detection include virus isolation, TEM for direct visualization of poxvirus particles, and WBS [108, 141, 146, 147]. Virus isolation is a critical method for confirming viral infections; however, MPXV isolation and cultivation require BSL‐3 containment and rely on cell lines (e.g., Vero, Vero‐E6, BSC‐1, HeLa) or chick embryos, restricting this method to reference laboratories [133, 148]. TEM enables direct visualization of characteristic brick‐shaped poxvirus particles but is limited by low throughput and specialized equipment requirements [133]. WBS for viral shedding has emerged as an indicator of community exposure. MPXV DNA has been detected in wastewater (e.g., the Netherlands, Italy), supporting its potential for population‐level monitoring [125]. However, the correlation between wastewater viral loads and clinical outbreak dynamics remains incompletely characterized, necessitating further longitudinal studies.

## 5 Mpox Vaccines

Vaccines stand as the most effective strategy for preventing infectious diseases. Currently, four vaccines have been approved for mpox and smallpox prevention: ACAM2000, MVA‐BN, LC16m8, and OrthopoxVac (Table 2). According to the WHO classification, ACAM2000 is categorized as a second‐generation vaccine, whereas MVA‐BN and LC16m8 are classified as third‐generation vaccines [149]. OrthopoxVac, developed through genetic engineering with the deletion of virulence‐associated genes, is designated as a fourth‐generation vaccine [32]. The approval of these vaccines underscores the availability of active intervention strategies for mpox prevention and control.

**TABLE 2:** Vaccines for mpox prevention.

Vaccines approved for mpox prevention					
Vaccine	Approval	Approval indications	Earliest approval date for mpox prevention	Dosage	References
MVA‐BN	US, European Union (EU), Canada (CA), and other regions	Indicated for prevention of smallpox and mpox disease in adults 18 years of age and older determined to be at high risk for smallpox or monkeypox infection	Jul 2013	Two doses, 4 weeks apart	[150, 151, 152]
ACAM2000	US	Indicated for active immunization for the prevention of smallpox and mpox disease in individuals determined to be at high risk for smallpox or mpox infection	Aug 2024	Single dose	[153]
LC16m8	Japan	Prevention of smallpox and mpox	Aug 2022	Single dose	[154]
OrthopoxVac	Russia	Prevent natural smallpox and other poxvirus infections among people aged 18–60	Nov 2022	Single dose	[155]

Selected mpox vaccines currently in clinical evaluation						
Vaccine	Developer	Vaccine platform	Clinical trial information			
			Title	Aiming	Phase/status	No.
BNT‐166A	BioNTech SE	mRNA vaccine	A Clinical Study Investigating the Safety and Immune Responses After Immunization With Investigational Monkeypox Vaccines	This is a dose‐escalation, Phase I/II study evaluating the safety, tolerability, reactogenicity and immunogenicity of the investigational RNA‐based multivalent vaccine candidate BNT166a for active immunization against monkeypox (mpox).	Phase I/II/active, not recruiting	NCT05988203
mRNA‐1769	ModernaTX, Inc.	mRNA vaccine	A Study to Investigate the Safety, Tolerability, and Immune Response of a Range of Doses of mRNA‐1769 Compared With Placebo in Healthy Participants From ≥ 18 Years of Age to < 50 years of Age	To assess the safety, tolerability and immunogenicity of mRNA‐1769 in healthy adult participants.	Phase I/II/completed	NCT05995275
MVA strain monkeypox live attenuated vaccine	Shanghai Institute of Biological Products Co. Ltd.	Attenuated live vaccine	A Study Evaluating the Safety and Immunogenicity of MVA Strain Monkeypox Attenuated Live Vaccine	To evaluate the safety and immunogenicity of receiving two doses of MVA strain monkeypox attenuated live vaccine in individuals aged 18 years and above.	Phase I/recruiting	NCT06771479; CTR20250074
Replication‐defective monkeypox vaccine	Beijing Institute of Biological Products Co. Ltd.	Non‐replication vaccine	—	—	Approved investigational new drug (IND)	—

*Source*: ClinicalTrials.gov website (https://clinicaltrials.gov) and China drug trials website (http://www.chinadrugtrials.org.cn).

### 5.1 MVA‐BN

MVA‐BN, a third‐generation live‐attenuated non‐replicating vaccine developed by Bavarian Nordic A/S, is administered as a two‐dose regimen with a 4‐week interval. It is indicated for use in individuals aged 18 years and older who are at high risk of smallpox or mpox infection (Table 3). Compared with traditional replicating vaccines, MVA‐BN exhibits significantly improved safety profiles, enabling its use in populations with compromised immune systems. In 2024, the WHO added MVA‐BN to its prequalification list as the first vaccine against mpox and designated it as a priority vaccine for mpox outbreak response [126, 156].

**TABLE 3:** Approval information of MVA‐BN vaccine.

Countries	Proprietary name	Approval date	Indications	Note	References
EU	Imvanex	Jul 2013	Active immunization against smallpox, monkeypox and disease caused by vaccinia virus in adults	Authorized for smallpox prevention in 2013, then expanded its indications to include mpox and diseases caused by vaccinia virus infections in 2020	[150]
CA	Imvamune	Nov 2013	Active immunization against smallpox, mpox and related orthopoxvirus infection and disease in adults 18 years of age and older determined to be at high risk for exposure	Authorized for smallpox prevention in 2013, then expanded its indication to encompass mpox and related orthopoxvirus infections and diseases in 2020	[151]
US	JYNNEOS	Sep 2019	Prevention of smallpox and mpox disease in adults 18 years of age and older determined to be at high risk for smallpox or monkeypox infection	In 2022, the Food and Drug Administration (FDA) further granted an emergency use authorization (EUA) for MVA‐BN, extending its use to individuals under 18 years of age and adding intradermal injection as an additional route alongside the previously approved subcutaneous administration	[152]

The MVA‐BN strain originates from the Modified Vaccinia Ankara (MVA) virus, a highly attenuated VACV strain derived from the Ankara vaccinia virus (CVA) through over 500 serial passages in chicken embryo fibroblasts (CEF) cells [157, 158, 159]. A key characteristic of MVA is its host restriction phenotype—an inability to infect and replicate in mammalian cells [160]. In 1998, Bavarian Nordic further passaged the MVA strain and renamed it MVA‐BN [161, 162, 163, 164].

The effectiveness of the MVA‐BN vaccine in the nonclinical phase is primarily supported by findings from MPXV challenge studies in nonhuman primates (NHPs). As detailed in FDA‐released documents related to the JYNNEOS application, a total of 11 preclinical studies were conducted in cynomolgus macaques using three primary MPXV challenge models (intravenous [IV], intratracheal, and aerosol) [152]. These studies demonstrated that MVA‐BN elicits the production of MPXV‐specific binding antibodies and neutralizing antibodies in macaques, thereby affording protection against severe mpox symptoms and mortality following MPXV challenge.

To date, over 20 clinical trials have been conducted to evaluate the efficacy and safety of MVA‐BN. A Phase III clinical trial (NCT01913353) directly compared MVA‐BN with ACAM2000 to assess the safety and efficacy of MVA‐BN in vaccinia‐naive healthy individuals. Peak plaque reduction neutralization test (PRNT) results demonstrated that two doses of MVA‐BN administered 28 days apart are non‐inferior to ACAM2000 in preventing smallpox in individuals with no prior smallpox vaccination history. Additionally, the incidence of adverse events (AEs), particularly Grade 3 or higher, in the MVA‐BN group during the two‐dose vaccination course was lower than that in the ACAM2000‐only group [152, 165]. In addition, given the risk of severe complications from replicating smallpox vaccines in immunocompromised individuals, Bavarian Nordic conducted Phase II trials in atopic dermatitis (AD) patients and in HIV‐infected individuals, respectively. Data from these studies supported the use of MVA‐BN in immunocompromised individuals [166, 167].

Following a comprehensive evaluation of the efficacy and safety of MVA‐BN, the US FDA approved the MVA‐BN vaccine for use in individuals aged 18 years and older at high risk of smallpox or mpox infection [152]. In 2022, the FDA granted an EUA for MVA‐BN in individuals under the age of 18, and also approved intradermal administration (one‐fifth of the subcutaneous dose) for adults over the age of 18 [152]. Currently, a subsequent Phase II trial (NCT05740982) in adolescents aged 12–17 years is ongoing, with interim analysis showing non‐inferior antibody titers compared to adults aged 18–50 and no serious vaccine‐related adverse events [168].

In real‐world settings, Mason et al. analyzed data from 16 MVA‐BN vaccine studies conducted from January 2022 to February 2024, reporting that for pre‐exposure prophylaxis, the estimated effectiveness of a single dose ranged from 35% to 86%, and two doses from 66% to 90%; for post‐exposure prophylaxis, the estimated effectiveness of one dose was 78% and 89% derived from two separate studies, respectively [169]. Similarly, Pischel et al. evaluated the effectiveness of third‐generation vaccines in studies spanning 1970–2023, with findings showing that the estimated effectiveness of one dose of MVA‐BN was 76% (95% CI: 64%–88%), and that of two doses was 82% (95% CI: 72%–92%) [170, 171].

MVA‐BN is now included in the US Routine Adult Immunization Schedule for any person at risk for mpox infection [172]. The most common adverse reactions following subcutaneous administration of MVA‐BN include solicited injection site reactions such as pain, redness, swelling, induration, and itching. For intradermal administration, common adverse reactions include injection site erythema, induration, pruritus, pain, fatigue, headache, myalgia, nausea, axillary pain, anorexia, arthralgia, chills, and axillary swelling. Importantly, adverse reactions observed in HIV‐infected adults and those with AD are comparable to those in healthy adults [152].

### 5.2 ACAM2000

ACAM2000 is a second‐generation live attenuated replicating vaccine derived from the conventional calf lymph‐derived smallpox vaccine Dryvax (Wyeth Laboratories, Inc.) [173]. Dryvax played a pivotal role in smallpox eradication across the Western Hemisphere and Africa but was limited by manufacturing processes that failed to meet modern regulatory standards, leading to substantial impurities and AEs [174, 175, 176, 177]. Concerns over Dryvax's safety, depleting stockpiles, and the imperative to address potential smallpox bioterrorism threats prompted Acambis, Inc. to collaborate with the US CDC in July 1999 to develop the cell culture‐based ACAM2000 [178, 179].

The master seed virus for ACAM2000 was generated by passaging material from three distinct Dryvax batches in MRC‐5 cells, followed by plaque purification, assessments of skin and neurotoxicity in animal models, and evaluation of attenuated phenotypes [178, 179]. Scaled‐up production was conducted in Vero cells, with subsequent concentration, purification, and lyophilization to form the vaccine bulk [179].

Initially developed for smallpox prevention, ACAM2000 underwent pre‐licensure safety and efficacy evaluations via head‐to‐head comparisons with Dryvax. Nonclinical efficacy assessments focused on immunogenicity profiling in monkey and mouse models, and protective efficacy against lethal vaccinia virus (WR strain) challenge in mice. Results showed that ACAM2000 induced comparable immunogenicity, neutralizing antibody levels, and T‐cell responses, as well as equivalent protective efficacy to Dryvax [178]. Safety evaluations encompassing rabbit skin toxicity (assessed by erythema and lesion diameters) and neurotoxicity in mice and monkeys (assessed by survival times) demonstrated that ACAM2000 exhibited lower toxicity than Dryvax [178, 180, 181].

During clinical development, Phase I–III trials enrolled vaccinia‐naive and previously vaccinated individuals, most of whom were in parallel‐group, double‐blind studies using Dryvax as the active control. Efficacy evaluations demonstrated that ACAM2000 induced robust cutaneous reaction rates and seroconversion rates, with non‐inferiority to Dryvax [40]. However, it elicited a slightly lower Day‐30 neutralizing antibody geometric mean titer (GMT) compared to Dryvax. Both GMT and seroconversion rates decreased with vaccine dilution—a phenomenon not observed with Dryvax [182]. In safety assessments, ACAM2000 recipients exhibited significantly less erythema at the vaccination site than Dryvax recipients [183]. Notably, cardiac AEs (myo‐/pericarditis)—previously associated with NYCBH strain‐based vaccines—were observed in both groups. The incidence rate was 5.73 per 1,000 vaccinations for ACAM2000, numerically lower than that in Dryvax recipients (10.38 per 1,000) but not statistically significant [175, 179, 183].

Following a comprehensive benefit‐risk assessment of these studies, the FDA approved ACAM2000 in August 2007 for active immunization against smallpox in high‐risk individuals aged ≥ 18 years [184]. The recommended regimen is a single dose, administered via percutaneous inoculation with 15 punctures using a sterile bifurcated needle—a technique specific to orthopoxvirus vaccination [153]. However, due to adverse reactions observed in clinical trials, a black box warning covering conditions such as myo‐/pericarditis, severe vaccinial skin reactions, accidental eye infection, and risks during pregnancy was included in the labeling of ACAM2000 [153]. As a live replicating vaccine, ACAM2000 is also contraindicated in immunocompromised individuals.

In response to the global mpox outbreak, in August 2024, the FDA expanded the authorized use of ACAM2000 to include mpox prevention under an Expanded Access Investigational New Drug (EA‐IND) designation [185]. This expansion was based on its established human safety data from prior use and findings from well‐controlled preclinical studies demonstrating protective efficacy against mpox [185]. Concurrently, ACAM2000 has been deployed in efforts to respond to mpox outbreaks across Africa. However, the WHO recommends ACAM2000 only for specific individuals following individual risk assessments, primarily when alternative vaccines are unavailable [126].

### 5.3 LC16m8

LC16m8 (also known as LC16 or LC16 KMB) is a third‐generation vaccine initially approved by Japan's Pharmaceuticals and Medical Devices Agency (PMDA) in 1975 for the indication of “prevention of smallpox” without age restrictions. In 2022, the PMDA extended its indication to include “prevention of mpox” [186]. The WHO has granted the emergency use listing (EUL) to the LC16m8 mpox vaccine, making it the second mpox vaccine endorsed by the WHO following the PHEIC declaration [187].

Derived from a natural variant of the Lister (Elstree) strain, LC16m8 exhibits high genetic concordance with the parental strain in the core genomic region [188]. Complete genome sequencing revealed that LC16m8 harbors a mutation in the immunogenic membrane protein *B5R* gene, which results in the lack of a full‐length B5R protein. Relative to its parental strain, LC16m8 exhibits favorable biological properties characterized by reduced virulence and enhanced safety [189].

To evaluate the efficacy and immunogenicity of LC16m8, a series of studies was conducted, including comparative assessments with Dryvax or the Lister strain. In the MPXV‐infected *Macaca fascicularis* model, LC16m8 demonstrated protective efficacy comparable to that of the Lister strain [190]. Similarly, in the BALB/c mouse model, LC16m8 and Dryvax exhibited equivalent immune responses [191]. Moreover, Iizuka et al. reported that a single dose of LC16m8 confers protection against MPXV for at least 1 year in *M. fascicularis* [192]. Following the global mpox outbreak, Kobiyama et al. conducted extensive immunological evaluations in mice (BALB/c, C57BL/6J, and CAST/EiJ) and NHPs, demonstrating that LC16m8 elicits robust immune responses across various preclinical models and further providing preclinical and early clinical insights into the efficacy and safety of LC16m8 against mpox [193].

In the clinical stage, a Phase I/II trial comparing the safety and immunogenicity of LC16m8 with Dryvax confirmed that the two vaccines have comparable safety profiles, with both inducing similar local and systemic reactions and no serious AEs or cardiac toxicity. Immunologically, both groups developed robust neutralizing antibodies against CPXV, MPXV, and VARV (titers > 1:40) with 180‐day immune memory; LC16m8 achieved 100% vaccination success rate versus 85% for Dryvax [194]. In addition, LC16m8 exhibits favorable safety and immunogenicity in healthy adults as well as well‐controlled HIV‐positive populations, and even shows a degree of protective efficacy in MPXV‐HIV coinfection patients. In healthy adults, LC16m8 induced seroconversion rates of 72%, 70%, and 88% against the MPXV ZR599 strain, MPXV Liberia strain, and LC16m8 strain by Day 28 (declining to 30%, 30%, and 76% by Day 168), with a 94% 14‐day vaccination success rate. While 98% of participants reported mild‐to‐moderate adverse reactions, no serious events or mpox cases occurred, confirming favorable immunogenicity and safety [195]. An open‐label randomized trial validated LC16m8's safety and immunogenicity in well‐controlled HIV‐positive populations, supporting its use in high‐risk groups despite unconfirmed efficacy in high‐incidence settings [196]. In post‐exposure prophylaxis research, a small study of six close contacts of mpox cases receiving LC16m8 showed no mpox development within 21 days, mild adverse reactions, and uneventful recovery of two appropriately treated HIV‐infected individuals within 28 days [197, 198].

Collectively, the aforementioned data demonstrate that LC16m8 exhibits favorable immunogenicity, protective efficacy, and an acceptable safety profile, with potential utility in post‐exposure prophylaxis. The WHO expert panel recommends its use in children and individuals at high risk of exposure, though it is contraindicated in pregnant women and immunocompromised individuals [199]. Ongoing clinical trials are further evaluating the mpox‐preventive efficacy of LC16m8 and conducting extended safety assessments [200, 201].

### 5.4 OrthopoxVac

OrthopoxVac, a fourth‐generation smallpox vaccine developed by the State Research Center of Virology and Biotechnology (SRC VB) VECTOR in Russia, was officially licensed in the Russian Federation in November 2022 for immunization against VARV, MPXV, and other orthopoxvirus infections [155]. The approval of this vaccine provides a new option for addressing the global mpox epidemic.

OrthopoxVac is a live‐attenuated vaccine based on the VACΔ6 strain, generated via targeted deletion of six key genes (*A56R, B8R, J2R, C3L, N1L, and A35R*) from the parental LIVP vaccinia virus. Maksyutov et al. employed PCR and sequencing analysis to show that after 15 passages in 4,647 cells, all viral DNA sequences remained unchanged, confirming the genomic stability of the VACΔ6 strain [202]. Notably, compared to the parental LIVP strain, the VACΔ6 strain exhibits significantly reduced reactogenicity and neurovirulence while exhibiting enhanced immunogenicity [203].

The licensing of OrthopoxVac is supported by a comprehensive portfolio of nonclinical and clinical studies demonstrating its efficacy in preventing smallpox and other orthopoxvirus infections [204]. In clinical practice, the vaccine is administered as a single 0.2 mL intradermal injection into the lateral third of the deltoid muscle. The vaccination response typically manifests as a distinct local skin reaction with progression from erythema and swelling to vesiculation, reaching maximal size between Days 8 and 10. Subsequently, the reaction resolves with scab formation, and upon scab detachment (typically by the third week), no residual scarring is observed [155].

OrthopoxVac is contraindicated in immunocompromised individuals and those receiving immunosuppressive therapy. The most frequent adverse reaction (incidence > 10%) is pain at the injection site. Less common adverse effects, observed in fewer than 1% of patients, include localized lymphadenitis, headache, malaise, fatigue, and elevated body temperature exceeding 37°C [155].

### 5.5 Other Potential MPXV Vaccines

Although approved mpox vaccines have demonstrated effectiveness, persistent concerns remain regarding the safety profile of live‐attenuated mpox vaccines, highlighting a critical opportunity for the development of novel mpox vaccine candidates. According to the WHO Mpox Vaccine Tracker, over 40 mpox vaccine candidates are currently in development, encompassing diverse technological platforms such as mRNA, subunit, and DNA vaccines. The majority of these candidates remain in the preclinical phase. Selected mpox vaccine candidates currently in clinical evaluation are summarized in Table 2.

BNT166 and mRNA‐1769 are mpox vaccines developed using mRNA technology and are currently in the Phase I/II clinical stage. BNT166 is a multivalent mRNA orthopoxvirus vaccine encoding MPXV antigens A35, B6, M1, and H3. In preclinical studies, it elicited robust antibody and T‐cell responses in murine and NHP models, and conferred protection against MPXV in virus challenge experiments [205]. mRNA‐1769 encodes MPXV surface proteins A35, B6, M1, and A29, and has been demonstrated to provide protection against MPXV challenge in NHP models. Notably, compared to MVA, mRNA‐1769 resulted in fewer lesions and reduced viral replication, while inducing enhanced neutralizing and functional antibodies [206].

The MVA strain monkeypox live attenuated vaccine's IND application was approved by the NMPA in September 2024, making it the first mpox vaccine authorized for clinical trials in China. This vaccine is currently in Phase I clinical trials (CTR20250074). The replication‐defective monkeypox vaccine was developed by deleting 26 gene fragments from the smallpox vaccine Tian Tan strain (VTT), rendering the vaccine unable to replicate in human cells and thereby enhancing its safety profiles [207]. Preclinical data demonstrated that this replication‐defective vaccine can successfully induce cross‐neutralizing activity against MPXV in mouse and rhesus monkey models [208]. JT118, a recombinant protein vaccine composed of the MPXV antigen A35 and M1, has also been approved by the NMPA for its IND application [209].

## 6 Treatments for MPXV Infection

Mpox, distinct from smallpox, is a self‐limiting disease, and the majority of cases are relatively mild; therefore, supportive treatments, including symptom management and rash care, are often adopted in clinical management for mpox patients [210, 211]. Notably, several specific antiviral agents initially developed for smallpox have been evaluated for their efficacy against MPXV. Herein, we systematically summarize the development history, efficacy, and safety profile of several key drugs, as well as the clinical trials conducted on mpox of these drugs (Table 4), along with the research progress of candidate compounds in the preclinical development stage.

**TABLE 4:** Main anti‐MPXV drugs.

Basic information				
Drug name	Structure	Mechanism of action	Dosage and administration	References
Tecovirimat (TPOXX/ST‐246)	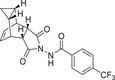	Interfering with the function of VP37 through specific binding	Capsules: 600 mg twice daily for 14 days; Intravenous injections: 200 mg twice daily for 14 days	[212, 213, 214, 215]
Cidofovir (Vistide/HPMPC)	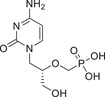	Inhibits viral DNA polymerase	Intravenous injections: 5 mg/kg once weekly, for 2 weeks; Topical: 1% cream applied locally	[216, 217]
Brincidofovir (TEMBEXA/CMX001)	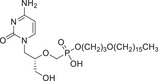	Inhibits viral DNA polymerase	Oral tablets and suspensions: 200 mg once weekly, for 2 weeks	[218]

Related mpox clinical trials						
Drug name	Title	Aiming	Phase/status	Study start	Results	No.
Tecovirimat	Study of Tecovirimat for Human Mpox Virus (STOMP)	To establish the efficacy of tecovirimat for the treatment of people with laboratory‐confirmed or presumptive HMPXV disease.	Phase III/active, not recruiting	September 12, 2022	According to an interim data analysis, tecovirimat did not reduce the time to lesion resolution or have an effect on pain among adults with mild to moderate Clade II mpox. No safety concerns were identified [219].	NCT05534984
	Tecovirimat for Treatment of Monkeypox Virus (PALM007)	To find out if tecovirimat is a safe and effective drug to treat monkeypox (mpox) in combination with standard of care (SOC).	Phase II/completed	October 10, 2022	Tecovirimat did not shorten the number of days to lesion resolution in patients with mpox caused by Clade I MPXV. No safety concerns were identified [220].	NCT05559099
	Assessment of the Efficacy and Safety of Tecovirimat in Patients With Monkeypox Virus Disease (UNITY)	To evaluate whether tecovirimat is an efficient and safe antiviral in the treatment of monkeypox in adults and adolescents (14 years old and older).	Phase III/recruiting	March 3, 2023	—	NCT05597735
	Tecovirimat in Non‐hospitalized Patients With Monkeypox (PLATINUM‐CAN)	The study will provide evidence on the efficacy and safety of Tecovirimat for laboratory‐confirmed monkeypox in outpatients with monkeypox infection and determine the feasibility of conducting interventional monkeypox trials in Canada.	Phase III/suspended	August 14, 2023	—	NCT05534165
	European Trial Into Mpox Infection (EPOXI)	To test the efficacy and safety of tecovirimat in patients with mpox (previously known as monkeypox) disease.	Phase IV/recruiting	August 9, 2024	—	NCT06156566
Brincidofovir	A phase III, multi‐country, randomized, placebo‐controlled, double‐blinded adaptive platform trial to assess the efficacy and safety of treatments for participants with monkeypox virus disease	The primary objective is to evaluate the clinical efficacy, as assessed by time to lesion(s) resolution in participants with mpox. The second objectives to evaluate the safety and efficacy, as assessed by mortality, hospitalization, complications, duration of symptoms in participants with mpox.	Phase III/recruiting	May 27, 2024	—	PACTR202405860723287

*Source*: ClinicalTrials.gov website (https://clinicaltrials.gov) and Pan African Clinical Trials Registry website (https://pactr.samrc.ac.za).

### 6.1 Tecovirimat

**FIGURE 3:**
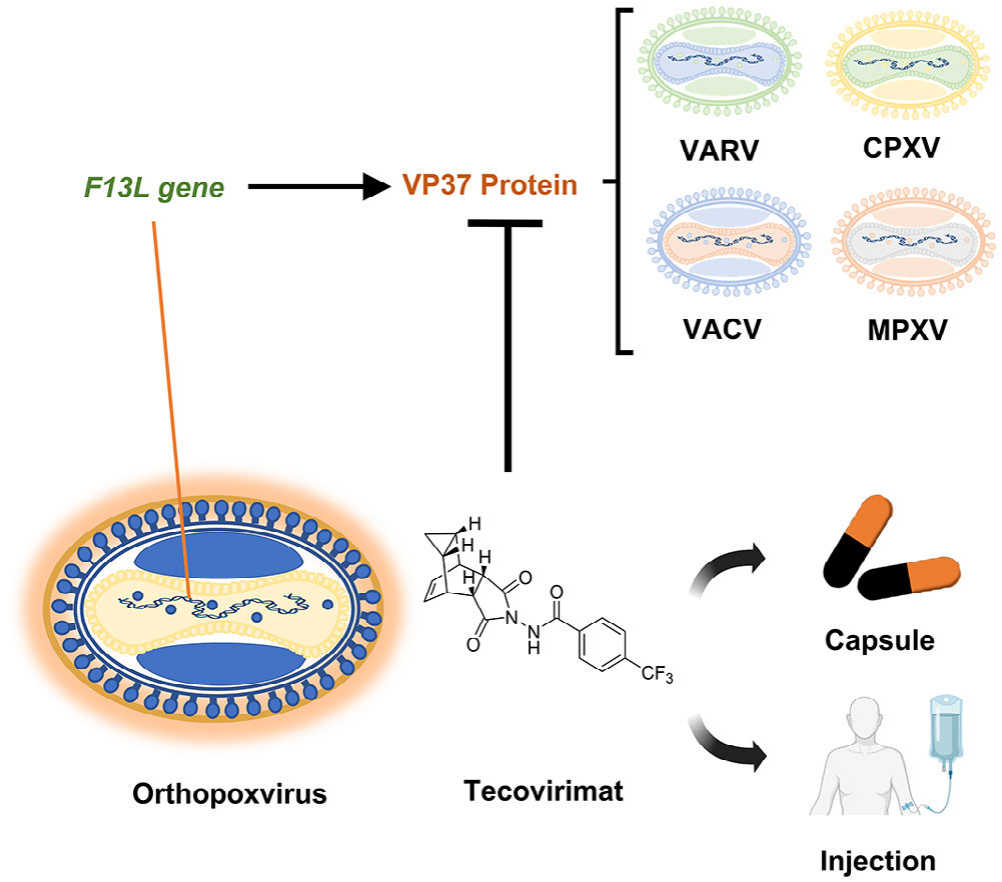
Tecovirimat exhibits broad‐spectrum antiviral activity against orthopoxviruses via targeting the VP37 protein. VP37, encoded by the *F13L* gene, is a highly conserved viral protein among orthopoxviruses and plays a critical role in the wrapping of IMVs within the TGN. By targeting the VP37 protein, tecovirimat inhibits the shedding and transmission of VARV, CPXV, VACV, and MPXV.

Tecovirimat is a tetracyclic acylhydrazide compound exhibiting broad‐spectrum antiviral activity against VARV, MPXV, and CPXV [221]. This molecule was screened from 356,240 compounds through in vitro anti‐VACV activity assays via high‐throughput drug screening in 2002 [222]. Further studies showed that tecovirimat induces the viral VP37 protein homodimerization and blocks the interaction of VP37 with cellular Rab9 and TIP47 proteins, preventing the formation of egress‐competent forms (i.e., IEV) of orthopoxviruses (Figure 3) [213, 214, 215].

TPOXX, the trade name of tecovirimat, has been developed and marketed by SIGA Technologies, Inc. in collaboration with the US Biomedical Advanced Research and Development Authority (BARDA), and is available in two dosage forms: oral capsules and an IV formulation [223]. Oral tecovirimat capsules are the first FDA‐approved specific therapeutic agents for VARV infections. As VARV was eradicated worldwide in 1980, clinical trials for tecovirimat capsules were primarily conducted in accordance with the FDA Animal Rule Guideline [215, 224].

Specifically, two key research components were conducted in the Phase II clinical trials to evaluate the clinical efficacy of tecovirimat capsules [225]. On the one hand, the fully effective protective dose of tecovirimat was investigated using two animal infection models: MPXV‐infected NHPs (NHP/MPXV model) and rabbitpox virus (RPXV)‐infected rabbits (Rabbit/RPXV model). With survival rate as the primary efficacy endpoint, the fully effective doses derived from two key pharmacodynamic (PD) studies (Nos. AP‐09‐026G and SR14‐008F) were 10 mg/kg in the NHP/MPXV model and 40 mg/kg in the Rabbit/RPXV model, respectively [225, 226]. Meanwhile, the pharmacokinetic (PK) characteristics, various treatment initiation times, and the minimum treatment duration of tecovirimat against virus infection using these animal models were evaluated and collected [226]. On the other hand, human dosing regimens that covered the exposure levels of a fully effective protective dose in animals were investigated in healthy volunteers [225, 227]. Based on data from these two components, the effective human dosage of tecovirimat was further estimated by establishing NHP and human population pharmacokinetic (Pop PK) models [225, 227].

Subsequently, a Phase III clinical study (No. SIGA‐246‐008) was conducted, in which researchers evaluated a dosing regimen of 600 mg twice daily for 14 consecutive days to validate tecovirimat's efficacy [228]. Comparison results showed that human exposure levels of tecovirimat were several times higher than those corresponding to the fully effective dose exposure levels in the NHP/MPXV model and the Rabbit/RPXV model [229]. Based on these PK results and safety data, the dosing regimen for tecovirimat capsules was defined as 600 mg twice daily for 14 days [212], and tecovirimat capsules were ultimately approved by the FDA for smallpox disease in 2018 [230].

Tecovirimat injection was developed based on the oral formulation and approved through the 505(b) regulatory pathway. SIGA Technologies, Inc. primarily compared the bioequivalence of the IV formulation and oral capsule formulation via clinical trial SIGA‐246‐IV‐202 and a Pop PK model [231, 232]. Overall, the IV dosing regimen of 200 mg, single infusion for 6 h, twice daily for 14 consecutive days [233], was demonstrated to be bioequivalent to TPOXX capsules in humans, supporting its market approval. It is now listed by the FDA and indicated for VARV infection (Table 5).

**TABLE 5:** Approval information of tecovirimat.

Dosage form	Country	Proprietary name	Approval date	Indication	References
Capsule	US	TPOXX	Jul 2018	Smallpox disease in adults and pediatric patients weighing at least 13 kg.	[230]
Capsule	CA	TPOXX	Nov 2021	Smallpox disease in adults and pediatric patients weighing at least 13 kg.	[234]
Capsule	EU	Tecovirimat SIGA	Jan 2022	Smallpox, mpox, cowpox, and complications due to the replication of VACV following vaccination against smallpox in adults and pediatric patients weighing at least 13 kg.	[235]
Capsule	the United Kingdom (UK)	Tecovirimat SIGA	Jun 2022	Smallpox, mpox, cowpox, and complications due to the replication of VACV following vaccination against smallpox in adults and pediatric patients weighing at least 13 kg.	[236]
Capsule	Japan	TEPOXX	Dec 2024	Smallpox, mpox, cowpox, and complications due to the replication of VACV following vaccination against smallpox in adults and pediatric patients weighing at least 13 kg.	[237]
Injection	US	TPOXX	May 2022	Smallpox disease in adults and pediatric patients weighing at least 3 kg.	[233]

Recently, TPOXX has been approved for mpox indication in the EU, UK, and Japan based on efficacy data from animal models, while in the US, clinical use of tecovirimat has been authorized under the EA‐IND protocol. Several studies have reported that most patients with MPXV infection experienced symptom improvement during tecovirimat treatment and achieved clinical recovery after a 14‐day treatment course [238, 239, 240, 241, 242, 243, 244, 245, 246]. However, it should be noted that most of the above studies lacked a control group, and thus, the definitive efficacy of tecovirimat in human mpox remains unconfirmed. The PALM007 trial was a randomized double‐blind placebo‐controlled study conducted in the DRC to evaluate the efficacy of tecovirimat against MPXV infection, and the results showed that the drug did not shorten lesion duration in patients infected with Clade I MPXV compared with the placebo group [220]. Similarly, interim analysis of another trial named STOMP revealed that tecovirimat did not reduce lesion duration in Clade II infections [219]. Nevertheless, secondary analyses from these trials indicated potential clinical benefits in mitigating severe disease progression and in patients administered during early infection stages [219, 247]. These collective findings underline the need for further investigation into tecovirimat's therapeutic potential.

Robust safety and favorable tolerability of TPOXX have been proven. The SIGA‐246‐008 trial revealed that tecovirimat capsules were generally well‐tolerated, with the most common treatment‐emergent AEs being mild in severity, including headache (12%), nausea (5%), abdominal pain (2%), and vomiting (2%) [225]. A retrospective analysis of 196 treated patients revealed similar patterns, with the most frequent adverse events being headache (6.1%), nausea (5.1%), and abdominal pain (4.1%) [248]. The PALM007 trial found comparable safety profiles between the treatment and control groups, with AEs reported in 72.9% of tecovirimat recipients versus 70.5% of placebo recipients [220]. The results from Phase I trials of TPOXX injection reported only adverse effects such as infusion‐site pain (associated with prolonged administration) and headache [231, 232].

### 6.2 Cidofovir and Brincidofovir

Cidofovir (CDV; trade name: Vistide), a cytosine nucleotide analog developed by Avet Pharmaceuticals Inc., was first approved by the FDA in 1996 for the treatment of cytomegalovirus (CMV) retinitis in HIV‐infected patients [249]. Beyond its original indication, CDV exhibits broad‐spectrum antiviral activity against a range of DNA viruses. Recent clinical studies and case reports have demonstrated its efficacy in treating infections caused by adenovirus (AdV) in transplant recipients, HSV infections, and varicella‐zoster virus (VZV) infections [250, 251, 252]. The antiviral mechanism of CDV involves intracellular phosphorylation to its active metabolite, CDV diphosphate (CDV‐PP), which is a competitive inhibitor of viral DNA polymerase, thereby preventing viral DNA synthesis and inhibiting viral replication [253].

CDV has emerged as a promising therapeutic candidate against MPXV due to its broad‐spectrum activity against DNA viruses, particularly against orthopoxviruses, both in vitro and in vivo [254, 255, 256, 257, 258]. CDV is available in two dosage forms: IV and topical. Systemic IV administration is a primary route for mpox patients and has shown potential therapeutic benefits. For example, Mondi et al. reported that four severe mpox patients in Italy treated with IV CDV exhibited rapid symptom improvement, achieving clinical recovery within 4–18 days [246]. In another study, an MPXV‐HIV coinfection patient showed accelerated lesion resolution and full recovery by Day 21 under treatment with CDV injection [259]. Clinical potential of topical CDV (1% cream formulated with Beeler base as an excipient) has been demonstrated in treating mpox skin lesions. A prospective study involving 24 mpox patients showed that topical CDV reduced the median lesion healing time to 12 versus 18 days in the supportive treatment group, and significantly decreased the positive rate of skin lesions (10% positive rate in the topical CDV group versus 62.5% positive rate in the supportive treatment group) [217]. It should be noted that the adverse effects and limited clinical data restrict the clinical application of CDV. Specifically, CDV exhibited dose‐dependent nephrotoxicity, which leads to proteinuria, glucosuria, elevated serum creatinine, and so forth [249, 260]. Consequently, renal function must be closely monitored during treatment. In addition, CDV has also been associated with other possible adverse reactions, including nausea, vomiting, neutropenia, elevated alanine aminotransferase levels, and localized burning sensation (with topical administration) [246, 261, 262].

To overcome the shortcomings of CDV, a long‐chain lipid‐conjugated prodrug of CDV, namely Brincidofovir (trade name: TEMBEXA; CMX001, BCV), was developed by Chimerix, Inc. in collaboration with BARDA. This structural modification reduces nephrotoxicity and markedly enhances oral bioavailability compared with CDV [263].

There are two dosage forms, including tablets and oral suspension, which were approved for the indication of smallpox by the FDA in 2021. Similar to tecovirimat, BCV was also developed under the FDA Animal Rules, and its efficacy was established using two animal models: the Rabbit/RPXV model and ectromelia virus (ECTV)‐infected mice (Mice/ECTV) model. With survival rate as the primary efficacy endpoint, the fully effective doses from two PD studies (Nos. CMX001‐VIR‐106 and CMX001‐VIR‐044) were 20/5/5 mg/kg (administered every 48 h for three doses) in the Rabbit/RPXV model and 10/5/5 mg/kg (administered every 48 h for three doses) in the Mice/ECTV model, respectively. Under these dosing regimens, lower mortality was observed in the treatment group compared with the placebo group [264].

According to the PK/PD data from animal studies, as well as PK parameters from clinical studies of healthy and non‐orthopoxviral‐infected subjects following oral administration of BCV tablets or suspension, Pop PK modeling was used to determine a final weight‐based dosing regimen. For general adult patients (≥ 48 kg), the recommended dose for both oral suspension and tablet formulations is 200 mg once weekly for 2 weeks (administered on Days 1 and 8) [264]. During the 2022 global mpox outbreak, BCV was included in the WHO list of potential therapeutic agents. Similarly, in the US, BCV became accessible for mpox treatment via an Investigational New Drug (e‐IND) application [210]. Recently, a Phase III, randomized, double‐blind, placebo‐controlled study, namely “MOSA,” is underway in Africa to assess the safety and therapeutic efficacy of BCV in mpox patients [265].

Regarding safety, BCV reduces the risk of nephrotoxicity compared to CDV, but may induce abnormal liver function, potentially affecting treatment tolerance in some patients [39, 263]. Thus, liver function should be regularly assessed during BCV treatment to prevent irreversible damage [2]. Other adverse reactions are generally mild to moderate. In Phase II and III clinical trials of BCV, common adverse reactions included diarrhea (8%), nausea (5%), vomiting (4%), and abdominal pain (3%) [264]. Additionally, the drug exhibits embryotoxicity and teratogenicity in animal studies; thus, it should be used cautiously in pregnant women and neonates [264].

### 6.3 Other Candidate Drugs

#### 6.3.1 Trifluridine

Trifluridine (TFT) is a thymidine nucleoside analog that exerts antiviral activity by inhibiting viral DNA synthesis. TFT exhibits potent inhibitory efficacy against MPXV infection in both cutaneous and ocular cell models, with additional activity against tecovirimat‐resistant virus strains [266]. It is reported that MPXV infection may give rise to various ophthalmic complications such as conjunctivitis and keratitis [266, 267]. Studies have shown that three of five patients who received tecovirimat‐TFT combination therapy showed improvement in their ophthalmic symptoms [268, 269]. Due to its poor corneal penetration and extremely low systemic absorption, systemic adverse reactions to TFT are rare, and common local adverse reactions include corneal inflammation, eyelid edema, and burning sensation [270].

#### 6.3.2 Tecovirimat Analog

Given the low solubility of tecovirimat, which may impact its pharmacological properties, researchers have focused on improving oral bioavailability through structural modifications.

NIOCH‐14, designated by the Novosibirsk Institute of Organic Chemistry, is a prodrug of tecovirimat that can be metabolized in vivo to exert anti‐orthopoxvirus activity. In the mouse model, NIOCH‐14 demonstrated 1.9‐fold higher absolute oral bioavailability than tecovirimat (22.8% vs. 12.1% at 50 mg/kg). Furthermore, NIOCH‐14 exhibited an excellent safety profile. Oral administration at 5 g/kg in mice caused no mortality or signs of toxicity, and both single‐dose and repeated‐dose studies in mice and rats showed no treatment‐related adverse effects on hematological or histopathological parameters [271]. NIOCH‐14 is currently in the Phase I clinical trial stage (NCT05976100). Wang et al. modified the aryl group of hydrazide in tecovirimat and generated a series of candidate compounds [272]. Several compounds exhibited anti‐VACV activity comparable to tecovirimat, with higher solubility (∼250 µg/mL at pH 2.0 and pH 6.8; tecovirimat: < 7 µg/mL) and a lower brain‐to‐plasma exposure ratio [272]. Shiryaev et al. reported a series of adamantane derivatives, which are similar to the cage structural moiety of tecovirimat in terms of space and lipophilicity. Among these, the best‐performing compound in vitro antiviral assays revealed that its half maximum inhibitory concentration (IC_50_) against VACV was 0.133 µM with a selectivity index (SI) > 2325; its IC_50_ against CPXV was 0.928 µM with an SI of 333, exhibiting potent antiviral activity and low cytotoxicity [273]. The above‐mentioned properties provide directions for future structural modification of tecovirimat.

#### 6.3.3 Nucleoside Analog

(*S*)‐HPMPA, a nucleoside phosphonate analog, exhibits good anti‐orthopoxvirus activity (IC_50_ ∼10 µg/mL) with a high therapeutic index in Vero and LC‐MK2 cells [254]. However, (*S*)‐HPMPA is hindered by low oral bioavailability and nephrotoxicity. Quenelle et al. reported two analogs of (*S*)‐HPMPA: ODE‐(*S*)‐HPMPA and HDP‐(*S*)‐HPMPA. In vitro assays demonstrated that both analogs were 65‐ to 300‐fold more potent against CPXV and VACV than (*S*)‐HPMPA. In vivo evaluations revealed that when administered orally at 30 mg/kg in CPXV‐ and VACV‐infected mouse models, both ODE‐(*S*)‐HPMPA and HDP‐(*S*)‐HPMPA reduced the mortality without observable toxic effects [274]. Additionally, HDP‐(*S*)‐HPMPA achieved significantly higher oral bioavailability (74%) compared to (*S*)‐HPMPA (24%) [274]. Zhang et al. reported three novel nucleoside analog prodrugs: BCV formate, ODE‐(*S*)‐HPMPA formate, and HDP‐(*S*)‐HPMPA formate. In vitro evaluations revealed that ODE‐(*S*)‐HPMPA formate exhibited superior antiviral activity against MPXV, with IC_50_ and IC_90_ values over 40‐fold lower than those of BCV. Moreover, all three prodrugs showed favorable safety profiles with half‐maximal cytotoxic concentration (CC_50_) values exceeding 40 µM, and no observable mitochondrial toxicity at 10 µM. In the VACV‐infected mouse model, ODE‐(*S*)‐HPMPA formate achieved the most promising therapeutic outcomes, demonstrating a >90% survival rate with minimal hepatotoxicity [275].

ASC10, an oral prodrug of molnupiravir developed by Ascletis Pharma, Inc., is converted to ASC10‐A‐TP in vivo, which binds to RNA‐dependent RNA polymerase and induces mutations in viral RNA, thereby inhibiting viral replication. The Phase I trial in healthy Chinese participants revealed that ASC10 was safe and well‐tolerated, exhibiting dose‐proportional exposure and minimal food effects [276]. A Phase Ib clinical trial is currently underway in the US for the treatment of mpox patients [277].

#### 6.3.4 Vaccinia Immunoglobulin Intravenous

Vaccinia immunoglobulin intravenous (VIGIV) is a polyclonal antibody preparation derived from the plasma of smallpox vaccine recipients, containing high‐titer antibodies against VACV [278]. It exerts its effect by neutralizing viral particles and inhibiting viremia [262]. In 2005, VIGIV was approved for the treatment of complications related to smallpox vaccination [279, 280]. During the 2022 global mpox outbreak, based on the antigenic cross‐reactivity within the orthopoxvirus genus, the US CDC implemented an extended access protocol allowing the use of VIGIV for the treatment of other orthopoxvirus infections, including MPXV [281, 282]. Accordingly, the US CDC recommended VIGIV for mpox patients with immune deficiencies, such as HIV‐infected individuals with CD4^+^ T‐cell counts below 350. However, further research is needed to verify its efficacy in MPXV infection [260].

#### 6.3.5 Other Reagents

Several compounds have also been reported to exhibit potential efficacy against MPXV infection. For instance, Gao et al. designed a series of peptidomimetic inhibitors targeting the MPXV core protease I7, with IC_50_ values ranging from 1.98 to 7.31 µM and CC_50_> 200 µM [283]. Zgarbová et al. reported a class of *S*‐adenosyl methionine (SAH) analogs as MPXV VP39 inhibitors, exhibiting IC_50_ values between 0.06 and 2.7 µM [284]. Chiem et al. screened the ReFRAME and NPC libraries for anti‐MPXV compounds and identified antimycin A, valinomycin, and rotenone as exhibiting excellent activity, with IC_50_ values of 0.01, 0.001, and 0.007 µM, respectively [285]. These compounds serve as promising lead scaffolds for the development of MPXV infection treatments.

## 7 Conclusion and Prospects

Since its isolation nearly 70 years ago, MPXV has diverged into distinct lineages with marked differences in virulence and transmission. Clade I (Ia/Ib) exhibits higher pathogenicity than Clade II (IIa/IIb), driven by genetic variations modulating host‐virus interactions [33]. While Clade I‐associated mortality (historically ∼10% in Africa) has declined due to viral virulence attenuation and improving clinical management, its persistent circulation (e.g., Clade Ia in the DRC) and Clade Ib's retained PHEIC status underscore ongoing global risks, amplified by cross‐border spread via travel. Though Clade IIb currently has low global prevalence, it previously caused a global outbreak and shows recent resurgence trends in selected regions (e.g., Sierra Leone). Notably, it disproportionately affects MSM, with mortality risks significantly elevated among those with HIV coinfection. In Uganda, 55% of MPXV‐related fatalities occurred in HIV‐positive individuals [46]. These divergent epidemiological patterns underscore the urgent need to advance understanding of pathogenesis, diagnostics, vaccines, and therapeutics.

To address these priorities, a systematic grasp of MPXV's fundamental virological characteristics is critical. Here, we review key features of virion morphology, genome organization, and lifecycle; however, significant gaps in virological understanding remain. For instance, the majority of studies on the poxvirus lifecycle are based on VACV, raising questions about whether MPXV employs unique viral mechanisms yet to be identified. Future research should prioritize functional conservation assessment between MPXV‐VACV homologous proteins through expanded homology‐based screening, coupled with systematic dissection of MPXV‐specific host interaction mechanisms. This will support the development of targeted antivirals and vaccines via novel strategies.

Beyond virological characterization, effective outbreak control hinges on robust diagnostic capacity. While the WHO has reported declining mpox cases in African regions, it emphasizes that actual cases may be undercounted due to gaps in diagnosis and surveillance [46]. Pragmatically, scarcity of diagnostic reagents and lack of POC rapid testing technologies hinder timely mpox detection, exacerbating transmission. Although RT‐qPCR remains the gold standard, its reliance on specialized infrastructure limits its utility in resource‐poor settings. Emerging isothermal amplification methods (e.g., LAMP) offer promising decentralized alternatives, with high sensitivity, operational simplicity, and minimal equipment requirements, supported by combinatorial diagnostic approaches. Accelerated clinical validation across diverse epidemiological contexts and equitable access initiatives are now essential to translate these technological advances from bench to field in high‐need regions.

Mpox vaccines serve as crucial public health tools for curbing the spread of MPXV, with their significance becoming particularly prominent amid the global spread of the epidemic in recent years. Currently, four vaccines have been approved for mpox prevention: ACAM2000 and MVA‐BN from the US, LC16m8 from Japan, and OrthopoxVac from Russia. ACAM2000, a second‐generation replicating smallpox vaccine, exhibits efficacy comparable to MVA‐BN but carries a higher risk of adverse reactions, including myo‐/pericarditis and severe vaccinial skin reaction [153, 165]. Consequently, it is only recommended when no alternative vaccines are available. In contrast, both MVA‐BN and LC16m8 are prioritized by the WHO [126]. MVA‐BN—a non‐replicating vaccine—stands out for its superior safety profile, making it suitable for immunocompromised individuals, and LC16m8 is approved for children over 1 year old. While these four vaccines mark a transition to proactive intervention, technical limitations and global health equity challenges persist. The landscape of mpox vaccine development is evolving toward next‐generation technologies and rational design strategies to address the drawbacks of live‐attenuated vaccines. For example, China's NMPA granted clinical approval to a replication‐deficient mpox vaccine derived from the VTT, which may provide a safe alternative for high‐risk populations in the future [207]. In addition, two mpox vaccines (mRNA‐1769 and BNT166) based on the increasingly promising mRNA technology are currently in Phase I/II clinical trials [205, 206].

Antiviral treatments play a vital role in the control of mpox and smallpox, particularly with drugs such as tecovirimat, CDV, and brincidofovir. Although tecovirimat is approved for mpox in the EU, UK, and Japan, its therapeutic efficacy still needs further research and evaluation. The PALM007 study, the first randomized controlled trial, was conducted to evaluate tecovirimat's safety and efficacy against MPXV infection [220]. Results showed no statistical significance for the primary endpoint: lesion resolution in Clade I mpox when comparing tecovirimat to placebo. Similarly, interim analysis of the STOMP trial (NCT05534984) revealed that oral tecovirimat did not reduce the time to lesion resolution compared to placebo in adults with mild‐to‐moderate Clade II mpox [219]. It is worth noting that these studies may have used less stringent eligibility criteria due to humanitarian considerations, so as to ensure that the maximum number of mpox patients receive treatment. For example, lesion counts of mpox patients in the PALM007 trial ranged from 1 to 10,264, potentially influencing the efficacy of tecovirimat due to variable disease severity. Nevertheless, the PALM007 trial demonstrated a strong safety profile for tecovirimat among all patients, and subgroup analysis suggested that early treatment or use in severe cases might offer clinical benefits [220]. These findings align with other reports. For instance, Aldred et al. observed that HIV‐infected patients treated with tecovirimat within 7 days had lower disease progression rates compared with those treated later or not at all [245]. Similarly, Karmarkar et al. found that in patients with severe disease, administering tecovirimat within 5 days of symptom onset resulted in faster symptomatic improvement compared with no tecovirimat treatment (−5.5 days; *p* = 0.04) [286]. Thus, additional clinical trials under refined research conditions are needed to further clarify the efficacy of tecovirimat against MPXV in humans, considering patient characteristics, disease course, health status, efficacy endpoint, and impacts of mpox genetic variants. Furthermore, combination therapy may improve MPXV treatment outcomes. For instance, in severe mpox patients with advanced HIV (CD4 < 200 cells/µL), early antiretroviral therapy, along with multipronged mpox treatment (e.g., tecovirimat combined with cidofovir or brincidofovir), may enhance efficacy, despite the risk of hepatotoxicity and nephrotoxicity, necessitating monitoring [287, 288].

To sum up, we systematically review recent advancements of MPXV research from five different perspectives, hoping that researchers gain insights from reviewing this article and develop more effective diagnostic methods, vaccines, as well as treatments to control and combat mpox.

## Author Contributions

Y.Z., W.Z., S.P., S.Y. and S.L. created the conceptualization. Y.Y., Y.S. and G.S. wrote the original draft. Y.Y., Y.S., G.S., C.N., X.Z., M.Z., T.L., S.Z., H.Z., A.L., S.Y., S.P., W.Z. and Y.Z. performed the review and editing. Y.Z., W.Z., S.P. and S.Y. provided supervision. All authors have read and agreed to the published version of the manuscript.

## Funding

This work was supported by grants from the National Key R&D Program of China (2022YFC2303300 to W. Z.).

## Ethics Statement

The authors have nothing to report.

## Conflicts of Interest

Author Yunzheng Yan, Yaqin Sun, Guangyan Sun, Cheng Niu, Xinyuan Zhao, Ming Zhao, Tongyao Liu, Suyue Zhang, Hui Zhai, Ankang Liu, Shouzhi Yu, Shuyuan Pan are employees in Beijing Institute of Biological Products Company Limited. Author Yuntao Zhang is an employee in China National Biotec Group Company Limited.The other authors have no conflicts of interest to declare.

## Data Availability

The authors have nothing to report.
